# Metabolic Biomarker Panels of Response to Fusarium Head Blight Infection in Different Wheat Varieties

**DOI:** 10.1371/journal.pone.0153642

**Published:** 2016-04-21

**Authors:** Miroslava Cuperlovic-Culf, Lipu Wang, Lily Forseille, Kerry Boyle, Nadine Merkley, Ian Burton, Pierre R. Fobert

**Affiliations:** 1 National Research Council, Ottawa, Ontario, Canada; 2 National Research Council, Saskatoon, Saskatchewan, Canada; 3 National Research Council, Halifax, Nova Scotia, Canada; Universita degli Studi di Pisa, ITALY

## Abstract

Metabolic changes in spikelets of wheat varieties FL62R1, Stettler, Muchmore and Sumai3 following *Fusarium graminearum* infection were explored using NMR analysis. Extensive 1D and 2D ^1^H NMR measurements provided information for detailed metabolite assignment and quantification leading to possible metabolic markers discriminating resistance level in wheat subtypes. In addition, metabolic changes that are observed in all studied varieties as well as wheat variety specific changes have been determined and discussed. A new method for metabolite quantification from NMR data that automatically aligns spectra of standards and samples prior to quantification using multivariate linear regression optimization of spectra of assigned metabolites to samples’ 1D spectra is described and utilized. Fusarium infection-induced metabolic changes in different wheat varieties are discussed in the context of metabolic network and resistance.

## Introduction

Fusarium head blight (FHB) is a fungal disease affecting different crops including major agricultural crops. FHB is mostly caused by fungal pathogen *Fusarium graminearum* Schwabe. FHB is becoming a major wheat disease in North America causing major losses in productivity. Even more importantly, the infected crop may contain significant levels of various mycotoxins which are highly hazardous to human and animal consumers [1]. FHB is currently treated with highly environmentally hazardous chemical fungicides that are effective only when used under specific conditions [2]. More resistant varieties and agricultural practices can help, however as none of the currently available varieties are fully resistant, these measures have been proven inadequate in severe epidemics. Thus novel, more resistant wheat varieties as well as innovative, non-toxic fungicides are sorely needed. While striving towards those ultimate goals it is also important to develop methods for quick and inexpensive detection of reduced susceptibility in wheat as well as early detection of infection in crop allowing targeted treatment planning. As plant and fungal metabolites play a major role in defense (reviewed in [3]) and virulence (reviewed in [4]), panels of metabolic biomarkers can provide information about resistance potential as well as initiation of infection.

Mass spectrometry (MS) metabolomics analysis of the effect of FHB in some wheat varieties has been presented by several groups. Hamzehzarghani et al. have explored metabolic differences between the FHB resistant cv. Sumai3, and the susceptible cv. Roblin 24 hours after Fusarium infection [5]. According the presented GC-MS measurements of metabolic changes following Fusarium exposure, a number of novel as well as known compounds are up-regulated by pathogen inoculation. Out of 49 metabolites upregulated following inoculation, 12 were observed only in the susceptible variety Roblin, and 13 are unique to the resistant variety Sumai3. Additionally, a subset of metabolites was shown to have different concentrations in Sumai3 and Roblin varieties prior to infection. This early study showed clear difference in metabolic response in relation to the susceptibility levels of wheat varieties following infection at one time point. A number of related publications followed and further showed significance of metabolism in plant response to Fusarium.

Kumaraswamy et al. [6] have investigated metabolic differences in five FHB resistant barley genotypes using an LC-ESI-LTQ-Orbitrap system. Fusarium inoculation resulted in the determination of metabolites that are resistance and pathogenesis related, and that can act as resistance indicators. Significant metabolites have been determined using t-test analysis with at least two-fold increase in abundance in resistant compared to susceptible lines. Resistance related compounds mainly represented phenylpropanoids, flavonoids and fatty acids. Significant concentration differences between resistant and susceptible lines have been determined for phenylalanine, p-coumaric acid, jasmonate, linolenic acid, total deoxynivalenol (DON) and DON-3-O-glucoside amongst others. Some of these metabolites can be perceived as possible biomarkers of enhanced resistance visible after the infection. At the same time, this study determined that major differences between resistant and susceptible varieties are in the activity of phenylpropanoids, fatty acid and flavonoid pathways with concentration differences in both precursors and products of these pathways.

Bollina and co-workers as well as Chamarthi et al. [7–9]have also investigated resistance related metabolites in FHB resistant barley lines. Metabolic differences between several FHB resistant and one susceptible barley line included 39 constitutive and 37 induced resistance related metabolites with higher concentrations in resistant compared to susceptible line [9]. These metabolites belonged to six chemical groups: phenylpropanoids, hydroxycinnamic acid amides, flavonoids, fatty acids, terpenoids and alkaloids. Differences were also observed in resistance related metabolite in distinct resistant lines suggesting possible biomarkers for marker assisted breeding.

Combined metabolomics and transcriptomics analysis of FHB infection in the model cereal species *Brachypodium distachyon* [10] have shown a major role of DON toxin in *F*. *graminearum* virulence. Additionally, transcriptomics analysis of plant genes showed induction of jasmonate and enthylene-signaling pathways as well as pathways involved in detoxification, metabolite transport and antioxidant and secondary metabolite production. Metabolomics analyses have shown increased production of tryptophan-derived metabolites as well as phenylpropanoids and phenolamines.

Recently, non-targeted metabolomics was used to, once again, investigate response differences in wheat varieties Sumai3 and Roblin to *F*. *graminearum* [11,12]. The resistance in Sumai3 was determined to be due to the accumulation of resistance related metabolites particularly ones belonging to the phenylpropanoid pathway causing reduction of pathogen advancement through increased host cell wall thickening and antifungal and antioxidant activity. Accumulation of resistance related metabolites in Sumai3 was reduced by trichothecene producting *F*. *graminearum*, possibly through trichothecenes/DON protein biosynthesis inhibition.

The majority of previously published studies utilized GC-MS or LC-MS for metabolomics experimentation due to their high sensitivity. NMR metabolomics, although less sensitive provides a ‘holistic view’ of the metabolome of biological systems [13]. In addition, NMR measurement can provide a more accurate quantification of a subset of metabolites with the possibility for identification of novel compounds from 1D and 2D spectra. NMR provides an accurate read-out of concentrations of a number of metabolites involved in primary metabolism including precursors for resistance related pathways as well as several secondary metabolites. NMR technique is by its nature non-targeted. Easy and inexpensive sample preparation and analysis make NMR a very attractive method for phenotype testing. The goal of this work was to determine whether NMR provides sensitive enough information to distinguish between different phenotypes as well as whether it is able to measure metabolic changes following *F*. *graminearum* infection. Quantification of obtained metabolic measurements provided biological indications of differences in more resistant or susceptible wheat varieties and suggested possible metabolic markers of resistance that can be translated to the field.

In this work we have developed a method for measurement, assignment and quantification of metabolites using NMR metabolomics. This method was used for investigation of metabolic profiles in four wheat varieties showing different levels of resistance to *F*. *graminearum*. Metabolic profiles were measured at two different time points following infection. Further identification of specific chemical factors and biological processes responsible for the differences in the susceptibility to FHB and toxin accumulation between varieties will aid in the development of high quality resistant wheat varieties [14].

## Materials and Methods

### Fungal material and inoculum preparation

*Fusarium graminearum* (isolate DAOM 180379 from the Canadian collection of fungal cultures, Ottawa, Ont.) was used in this study. Fresh inoculum was taken from the stored type culture at monthly intervals. For the production of macroconidia, a plug of actively growing *F*. *graminearum* was placed in the center of a Petri dish containing Soft Nutrient Agar (SNA). Plates were placed under a combination of fluorescent and UV lights for 5 days at 23°C. Macroconidia were harvested by pouring a small amount of sterile water over the culture in the dish and by either gently scraping the surface with a bent glass pipette or washing with a gentle stream of water, using a pipette. A working concentration of 10^5^ macroconidia/ mL was used for inoculation.

### Plant material and infection procedure

All experiments were conducted in the environment-controlled growth chamber. Canadian germplasm ‘FL62R1’ was developed by Dr. André Comeau and Francois Langevin (AAFC- St. Foy) [15]. Canadian germplasm ‘Stettler’ and ‘Muchmore’ were developed by Drs. Ron DePauw and Richard Cuthbert (AAFC-Swift Current) [16,17]. Wheat seeds were sown in peat pots (diameter, 12.7 cm) and maintained in a growth chamber at 21°C/19°C:day/night cycle, with 16 h of light per day until flowering.

At mid-anthesis, single floret inoculation with the *Fg* strain was carried out by pipetting 10 μl of the macroconidia suspension (10^5^ per ml) between the palea and lemma. Inoculated plants were incubated in a dew chamber for 2 days before returning to the growth cabinet for the remainder of the experiment. For disease severity tests, a pair of alternate spikelets in the middle of head was inoculated. The first visual disease symptoms in infected rachis nodes appeared between 5 and 6 days after inoculation (dpi). Accordingly, the number of infected rachis nodes from the inoculated site was recorded shortly thereafter, at 7 dpi, and at weekly intervals thereafter, until heads of susceptible varieties were completely bleached (21 dpi). Disease severity was indicated as the percentage of number of infected nodes in total number of nodes in a head. Two heads per plants and 20 plants per variety, for a total of 40 heads per variety per time point, were examined. This experiment was repeated three times. Data in each time point were statistically analyzed using a one-way Analysis of Variance (ANOVA), General linear Model (SAS Institute Inc., http://www.sas.com) and significance between different variety was determined by post-hoc test (Tukey’s Honest Significant Difference test).

For metabolomics experiments, 10 spikelets midway along the spike were point inoculated with 10 μl of the macroconidia suspension as described above. At 48 and 96 hours post inoculation (hpi), infected spikelets and rachis were dissected and collected separately. Three heads from each variety were pooled as one biological replicate and five biological replicates were harvested per time point per variety. Samples were ground with liquid nitrogen and dried in a freeze dryer for 72 hours. Lyophilized powder samples were weighed and divided into two aliquots of 100 mg. Two ml of methanol:water solution (40:60 v/v) was added, mixed for 10 min using a minimix standard shaker, vortex and sonicated for 15min at room temperature, and centrifuge for 20 min at 3500 g. The supernatant was decanted and dried under gas N2 at room temperature.

The time points for metabolomics analysis (48 and 96 hpi) were selected based on a number of criteria to provide the most informative metabolic markers. The outcome of the interaction between wheat and *Fg* relies predominantly on the early infection process [18]. Given that *Fg* is necrotrophic pathogen, the host needs to induce defense responses as quickly as possible before cells are killed by mycotoxins [18]. According to many earlier reports (e.g. [19–21]) and our unpublished observations, *Fg* spores can germinate and colonize on the host tissue within 1 or 2 days after pipette inoculation. After 3–4 days, hyphae attempt to penetrate into the rachilla tissue between the spikelet and rachis and fungal toxins begin to accumulate [21]. Accordingly, the most active phase of infection process occurs from 2 dpi to 4 dpi, which influenced our selection of time points for metabolomics analysis. This is also in agreement with time period chosen in similar studies that have revealed dynamic changes in the metabolome [5,7,8,11].

### NMR experimentation

The residue obtained after drying was dissolved in 0.6 mL of deuterium oxide with added 100 mM phosphate, keeping the sample at pH 7.2 and 0.1mM TMSP reference. These samples were pipetted into a 5-mm NMR tube for NMR analysis. All ^1^H NMR measurements were performed on a Bruker 700 MHz spectrometer at 298 K. 1D ^1^H NMR were measured for all samples using 1D NOESY water suppression sequence. 2D JRES pulse sequence follows recommendation made by Parsons et al. [22]. Due to longer acquisition time 2D JRES spectra were measured only for one representative control sample for each studied wheat variety. For the same samples we also measured 2D TOCSY spectra. 2D JRES and 2D TOCSY spectra were used for assignment of measurable metabolites. All 1D and 2D spectra were processed using MestReNova 9.1.0 software. Spectral preprocessing for 1D spectra included: exponential apodization (exp 1); global phase correction; and normalization using the total spectral area. Spectral regions from 0.5–9.5 ppm were included in the normalization and analysis. 2D spectra were processed using standard procedure recommended in the MestReNova documentation.

### Data analysis

Data analysis procedure is outlined in S1 Fig and includes both qualitative and quantitative analysis approaches. Data pre-processing including data organization, removal of undesired areas, normalization, as well as data presentation was performed with Matlab vR2010b (Mathworks). Minor adjustments in peak positions (alignment) between different samples were performed using in-house developed alignment software (http://gast.nrcbioinformatics.ca) as well as a publicly available method Icoshift for automatic spectral alignment [23]. Principal component analysis (PCA) was done using the Matlab platform and hierarchical clustering was performed with TMeV software. Correlation analysis and network presentation was performed using Matlab. Feature selection was done with the Significance analysis for microarrays (SAM) method [24] as provided in TMeV.

Metabolite assignment was performed using 2D JRES and TOCSY data. 2D JRES spectra were assigned using database of 2D JRES spectra and automated assignment method provided under Birmingham Metabolite Library [25]. Assignment of peaks in the TOCSY spectra was performed using Madison Metabolomics Consortium Database and tools [26]. Total of 60 metabolites were included in analysis and include molecules that were present in both TOCSY and 2D JRES spectra as well as the once present in either TOCSY or 2D JRES but had distinguishable peak in the 1D ^1^H spectra.

Spectra for 60 metabolite used in quantification of 1D 1H spectra were obtained from the Human Metabolomics Database (www.hmdb.ca) or Biological Magnetic Resonance Databank (www.bmrb.wisc.edu), processed using MestReNova 9.1.0 software. Spectral preprocessing for standards spectra included: exponential apodization (exp 1); global phase correction; and normalization using the total spectral area. Spectral regions from 0.5–9.5 ppm were included in the normalization and analysis. Prior to quantification standard spectra were aligned to sample spectra using peak alignment by fast Fourier transform cross-correlation [27]. An automated method for quantification based on multivariable linear regression of spectra with appropriately aligned standard metabolite data from databases was developed previously [28] and utilized in this study. The assumption behind this approach is that the spectrum of a mixture is the same as the combination (sum) of spectra of individual components measured under the same conditions. Here relative metabolite concentrations were estimated using nonlinear curve-fitting with the multivariate least-squares approach. The partial least square regression analysis result was used as the starting point and the model was constrained to concentrations greater than or equal to zero. The deconvolution of spectra of mixtures, such as in metabolomics, with many strongly overlapping lines, possibly with an unknown number of lines and atomic groups, each with a different line width is extremely difficult and thus it is important to determine an optimal solver for this problem. Generally, the solution is found by minimizing the square root of difference between the model and the real spectrum. The best result, i.e. the model with a minimal error was obtained with Levenberg-Marquardt curve fitting and this method was used for quantification of metabolic data used in further analysis. The Levenberg-Marquardt method is specifically designed for solving non-linear curve-fitting problem in a least-squares sense and additionally allows inclusion of a constraint that concentrations have to be non-negative. Quantification error is estimated by performing the multivariate linear regression analysis as described above but with one metabolite at a time removed from the analysis. In this way, we are estimating errors caused by omitting metabolites from the analysis and the uniqueness of spectral features for metabolite quantification. Values for metabolite concentrations and the errors can be obtained by request from the authors. Relative, average concentration of metabolites in different samples with standard deviation across biological replicates is provided as S1 Table.

## Results and Discussion

**Fig 1:**
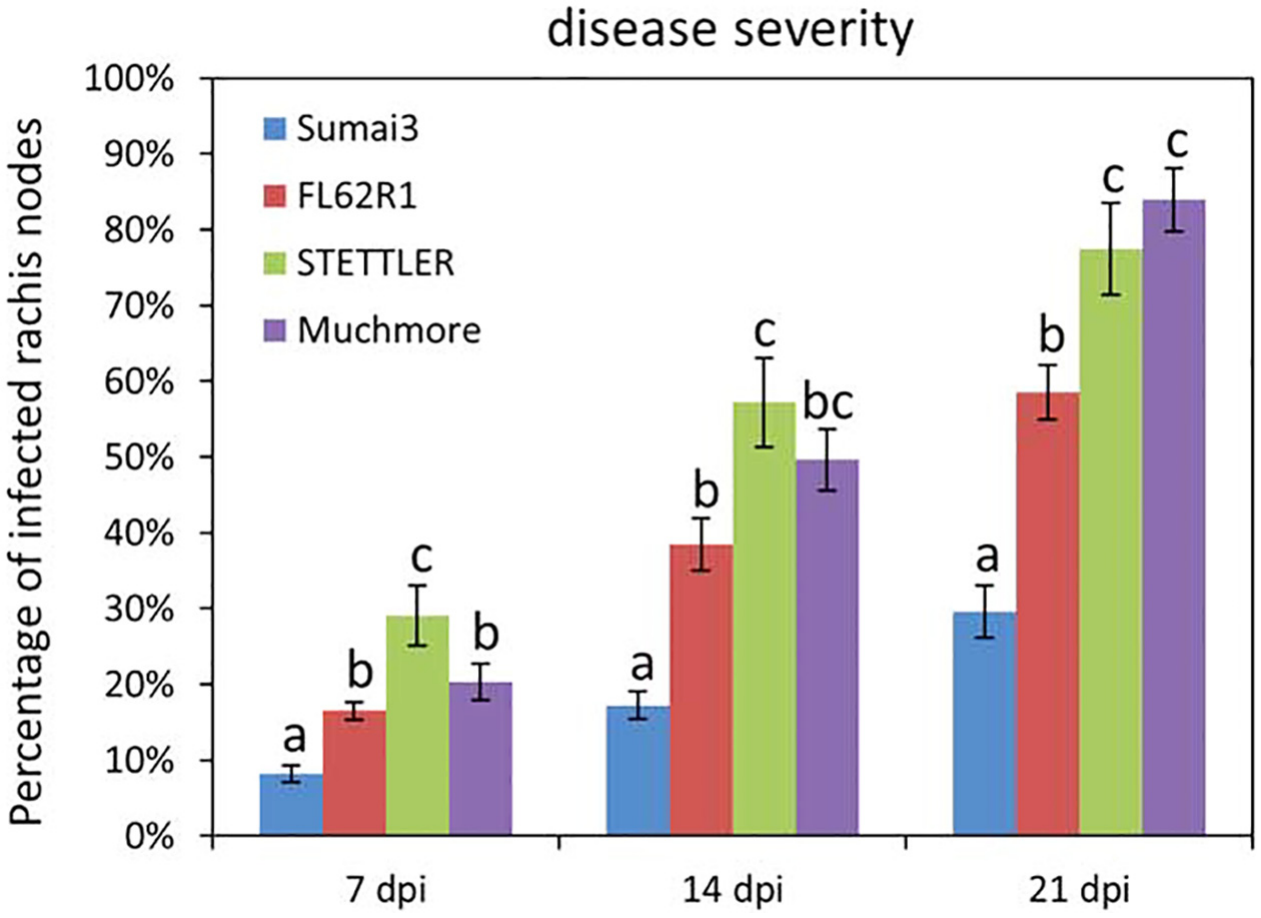
Disease severity levels in four wheat varieties: Sumai3, FL62R1, Stettler, andMuchmore. The percentage of infected rachis nodes per headwas scored at the time points indicated. Two heads per plant and 20 plants per variety, for a total of 40 heads, were examined for each variety at each time point.Values represent means ± standard error. A one-way ANOVA of data were performed in each time point at α = 0.05 to determine significance among different varieties. Histograms with different letters are statistically different.

Resistance to FHB is complex process, consisting of resistance to the initial infection (type I resistance) and to the subsequent spread of fungus through infected spikes (type II resistance) [29]. The focus of the present study is to identify metabolomics changes associated with type II resistance. Ideally, germplasm exhibiting different levels of type II resistance would be best suited for this analysis. To this end, FHB disease severity on four varieties (Sumai3, FL62R1, Settler and Muchmore) was assessed following point inoculation of a pair of spikelets in the middle of wheat heads with *Fusarium gramminearum* (*Fg*). The number of infected rachis nodes was scored at different time points after the onset of visual disease symptoms. The spread of fungal growth in Sumai3 was significantly lower than in the three other varieties at all time points (Fig 1). The spread of *Fg* in FL62R1 was significantly lower than in Stettler at 14 and 21 dpi and significantly lower than in Muchmore at 21 dpi (Fig 1). Similar results were obtained in two additional independent experiments (S2 Fig). Thus, among tested varieties, Sumai3 displayed the level of highest type II FHB resistance, with FL62R1 having moderate resistance, and Stettler and Muchmore being more susceptible.

To understand the molecular mechanism of FHB resistance, metabolomics profiling analysis was performed on these four varieties. Spikelets were inoculated as for the disease testing, and samples collected and processed for NMR analysis. Tissue displaying visible disease symptoms, while desirable to assess type II resistance, are dead or dying from the effects of mycotoxins and not informative for identifying plant defense-related metabolites. Here we selected 48 and 96 hours post infection (hpi) as sampling times as these correspond to the most active and determinative phase of the interaction between *F*.*g*. and wheat heads, between 2 dpi to 4 dpi [19,20,21]. This time range has been shown previously to involve dynamic changes in the metabolome [5,7,8,11].

**Fig 2:**
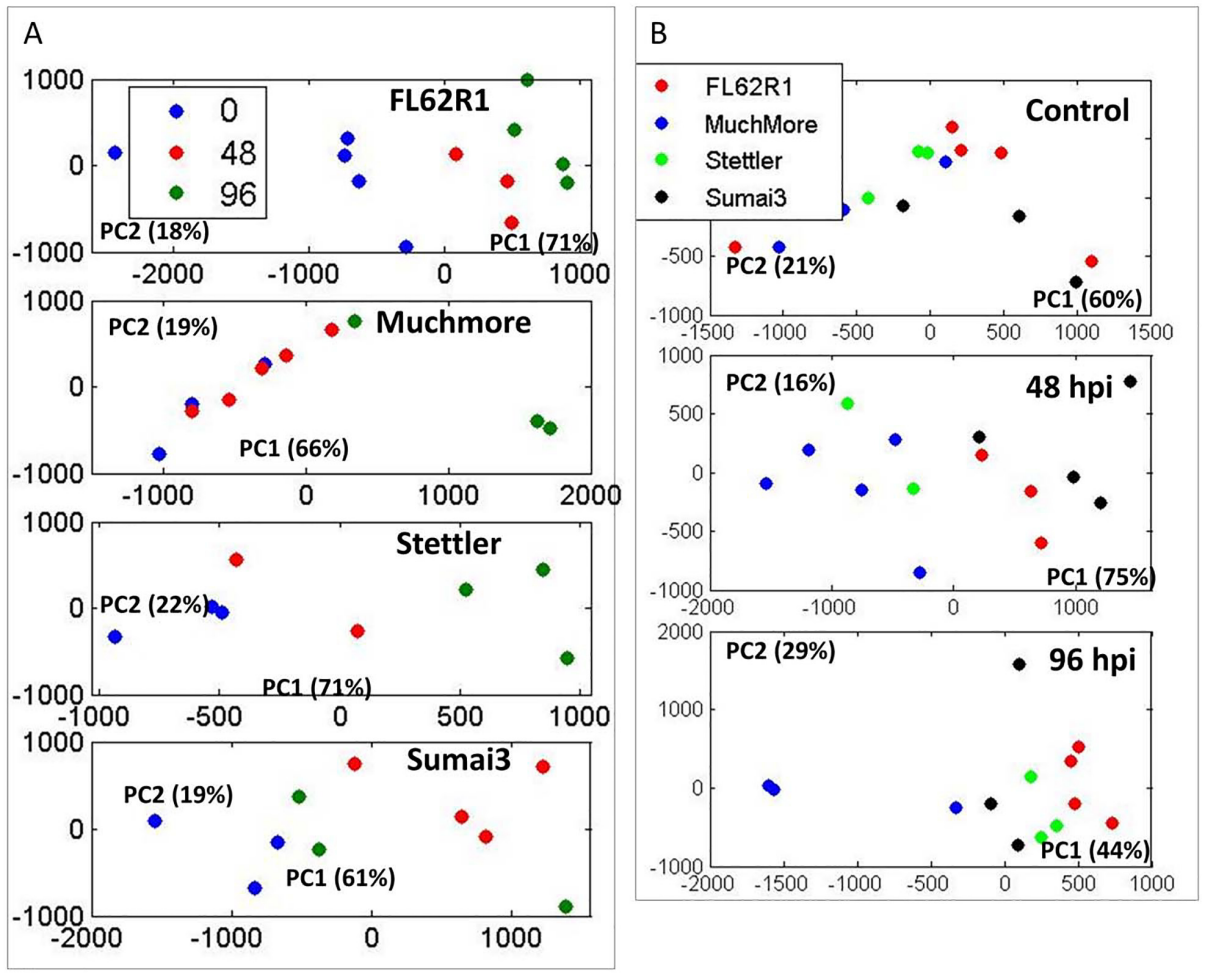
PCA of 1D 1H NMR spectra for four wheat varieties at different time points. A. Analysis of profiles for each wheat type separately showing changes in metabolic profile at 0, 48 and 96 hpi. B. Analysis of metabolic profile differences between wheat varieties at different time point.

All spectra are shown in S3A Fig following spectral processing and alignments using the Icoshift method running under Matlab [23]. Principal component analysis of complete 1D spectra show major variances and trends in the data. Fig 2A shows PCA for samples from each wheat variety at three different time points—control and 48 and 96 hpi. Distinct relative change between different varieties is apparent. FL62R1 and Stettler show increased metabolic changes following later infection time points. No major metabolic changes appear to be in Muchmore variety at 48 hpi, but at 96 hpi profile change is apparent at the PC1 level. Sumai3, the variety with the highest level of resistance, shows a major metabolic profile shift at 48 hpi but then at later time the metabolicprofile appears to revert back to the control levels. When exploring the difference between 4 varieties at different time points (Fig 2B) there is no significant difference in PC1 or PC2 in the control state, however at 48 hpi the metabolic profile of the most resistant varieties, FL62R1 and Sumai3 becomes clearly distinct in PC1. At 96 hpi metabolic profiles of all varieties are weakly separated with the largest difference between FL62R1 and Muchmore varieties.

**Fig 3:**
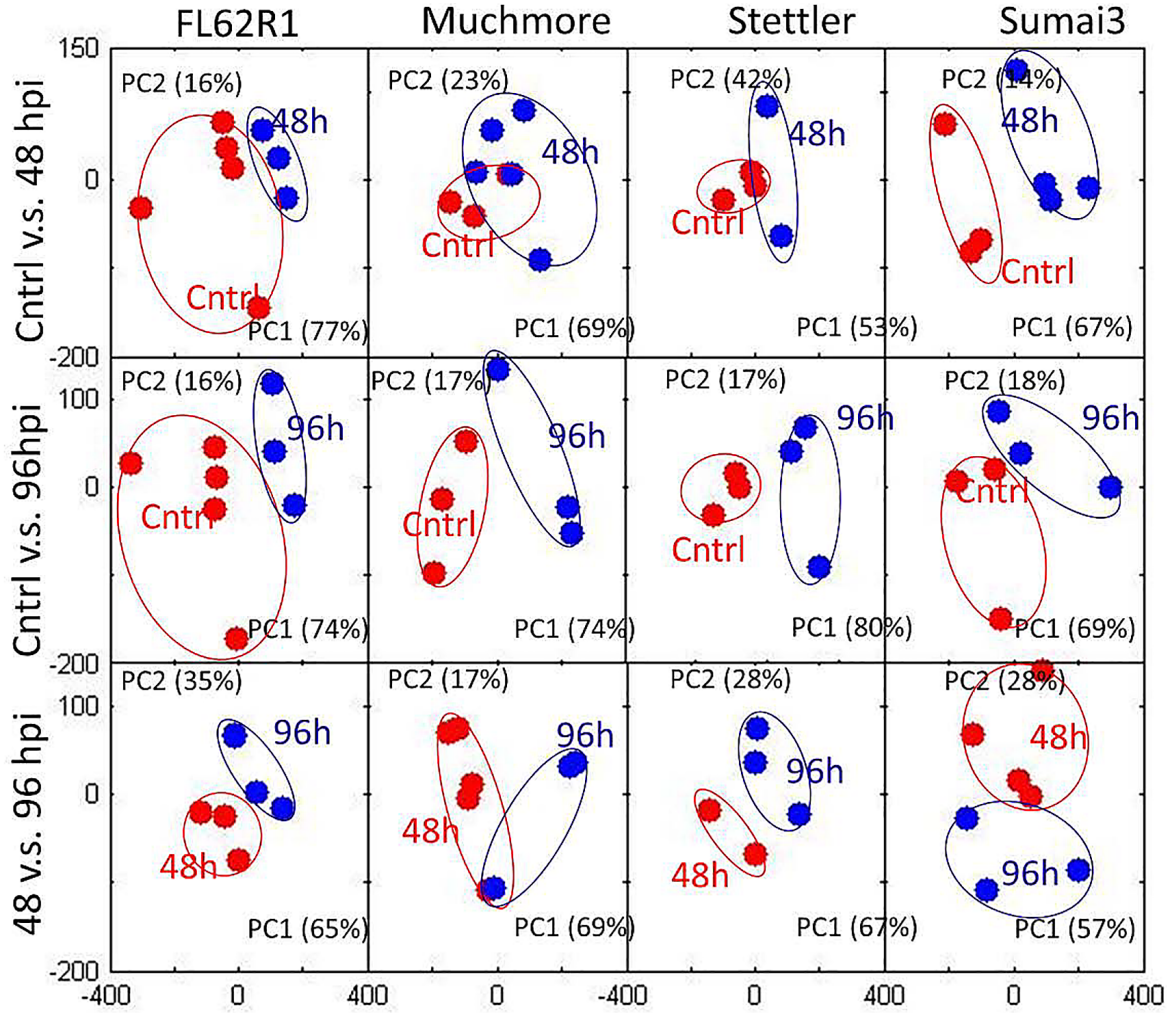
PCA of 1D 1H NMR spectra for four wheat varieties at different time points showing major profile differences between individual time points for each wheat type.

When looking at individual varieties (Fig 3), samples obtained at different time points are co-clustering particularly for FL62R1 between control and individual treated groups. In Sumai3, particularly strong separation is visible between control and 48 hpi and for Stettler there is some clustering of control and 96 hpi samples.

**Fig 4:**
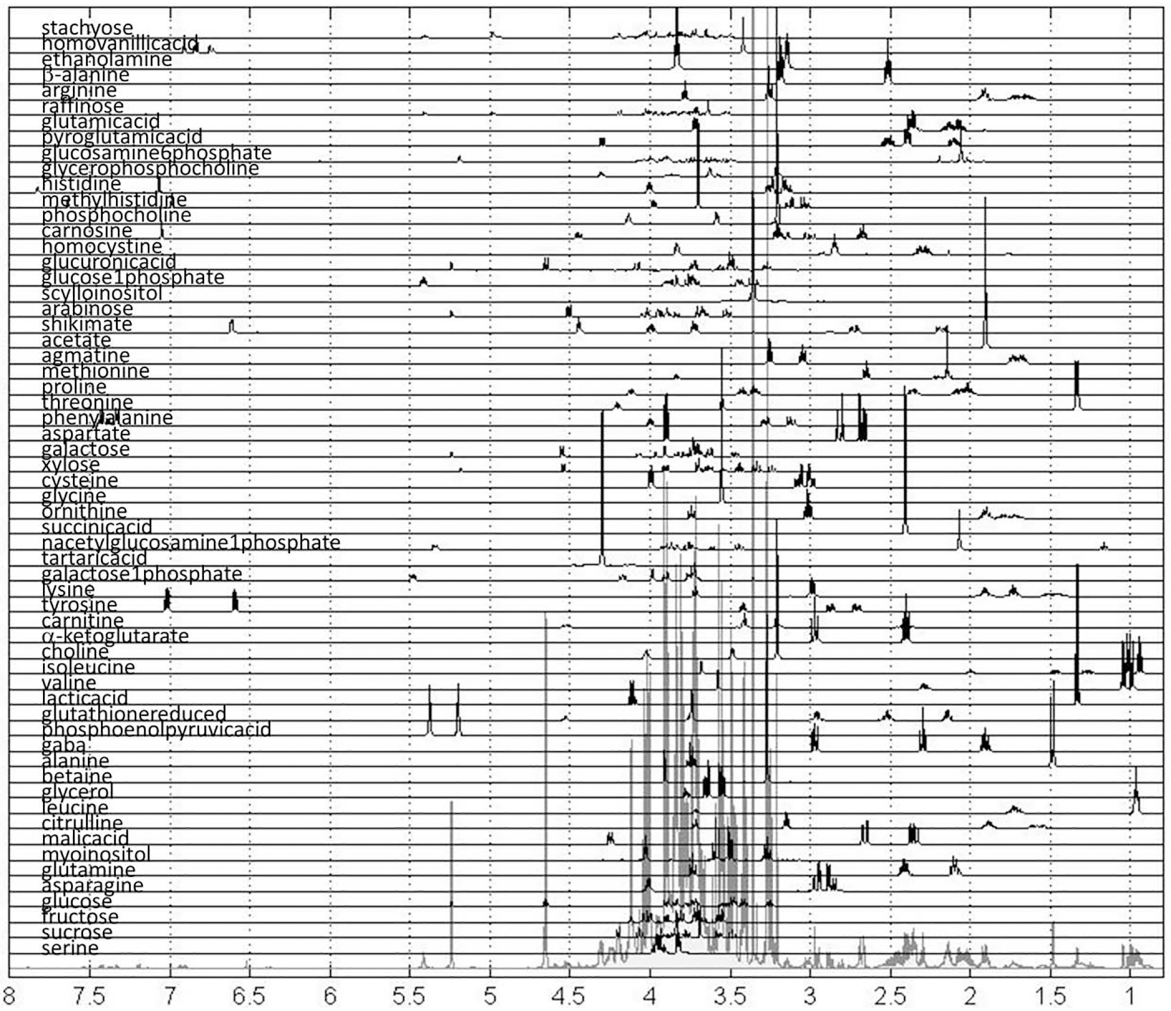
Spectra of standards used in the quantification analysis.

For one replicate of each variety in the control set we have also measured TOCSY and 2D JRES spectra and these data were used for metabolite assignment (S4 and S6 Figs). 2D spectra were used for assignment of measured metabolites using tools and methods described above. Spectra for all 60 assigned metabolites following preprocessing and alignment are shown in Fig 4.

**Fig 5:**
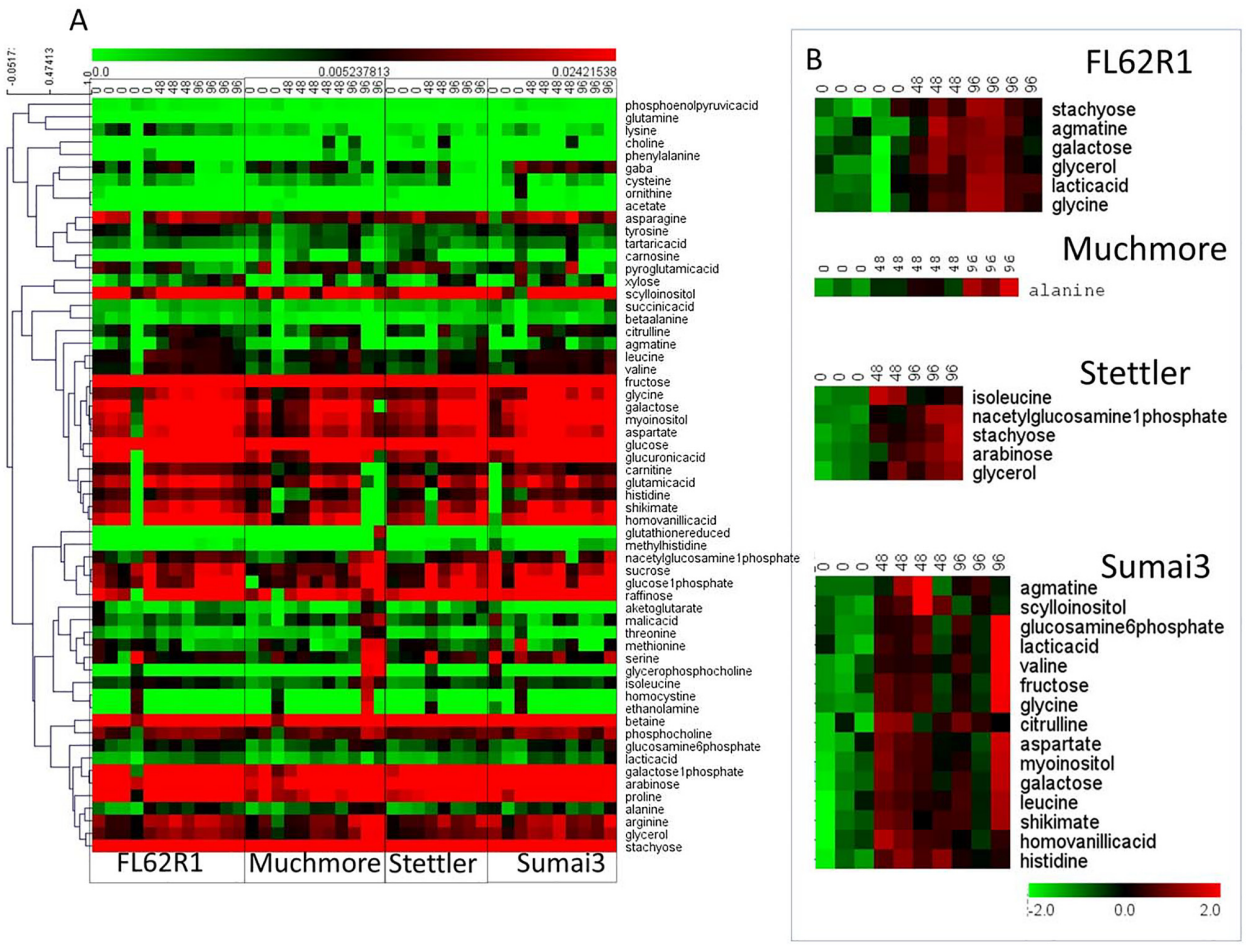
A. He at map representation of hierarchically clustered quantitative metabolic data for 60 metabolites assigned from 2D spectra and quantified in all 1D 1H NMR spectra. The obtained values are shown following scaling of spectra but without any scaling or normalization of metabolite concentrations. B. Significant analysis of microarray (SAM) analysis of major concentration changes between control and 2 treated groups separately in four wheat subtypes following sample and metabolite scaling.

These spectra have been utilized as standards for quantification using methods described in the Materials and Methods and used previously [28,30,31] resulting in the values presented as a heat map in Fig 5A.

**Fig 6:**
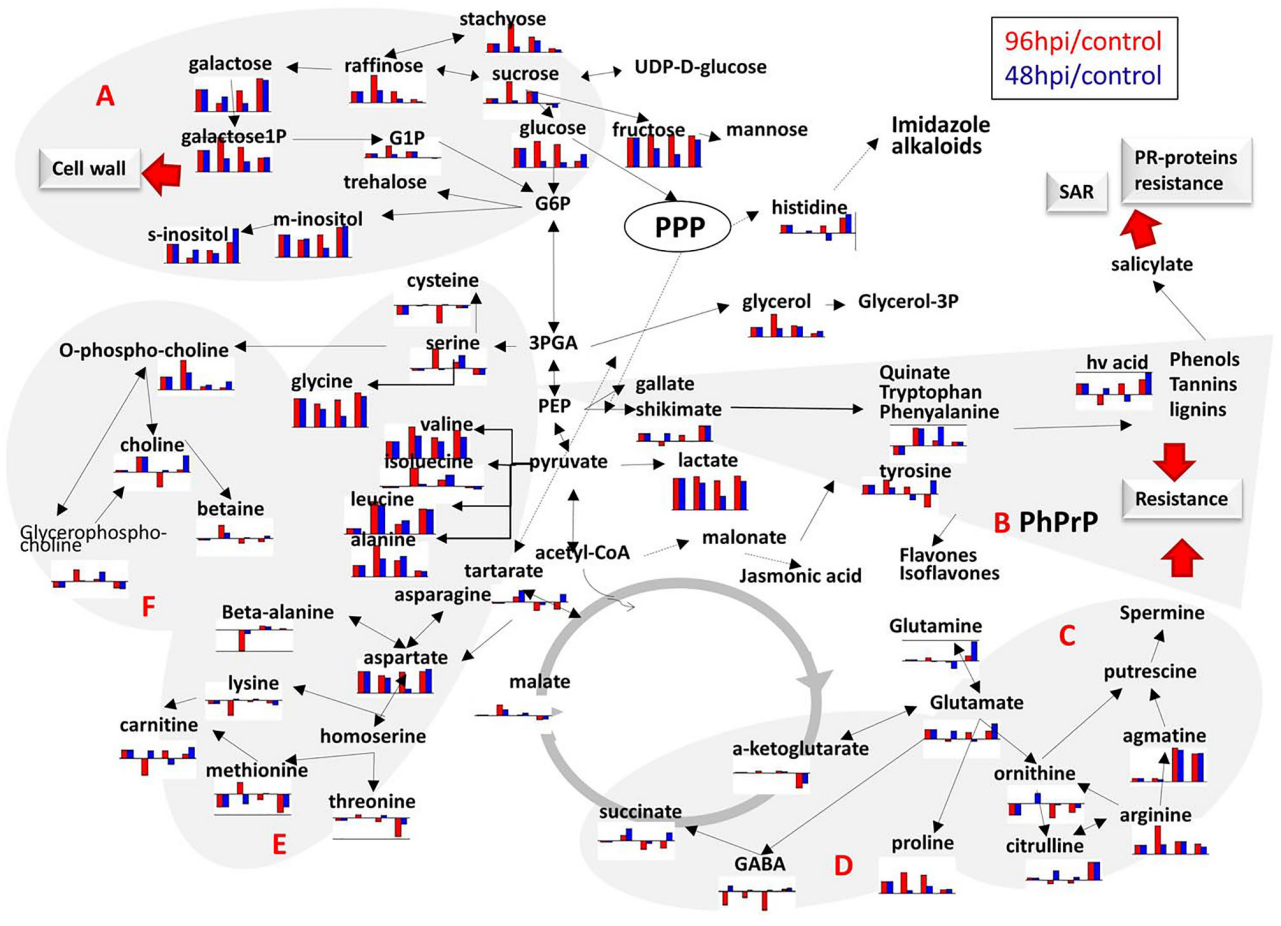
Schematic overview of metabolites measured in the presented experiment and their significance in resistance response through metabolism. Histograms indicate logarithms of concentration change at 48hpi (red) and 96hpi (blue) relative to the control for (in order): FL62R1, Muchmore, Stettler and Sumai3. In plot zero value (no change from control) is shown with the thick line. Positive values indicate concentration increased and negative concentration decrease relative to the control values. A. Metabolites involved in cell wall development. B. Phenylpropanoid Pathway (PhPrP) resulting in production of active phenolic phytochemical known as important factors in plant resistance to pathogens. C. Spermine biosynthesis pathway. Spermine has been hypothesized to act as an inducer of PR proteins and as a trigger for caspase-like activity (Crampton et al., 2009); D. gamma-Aminobutyric acid (GABA)–shunt block bypassing two steps in tricarboxylic acid (TCA) cycle and leading to production of GABA from glutamate; E. Amino acid metabolism; F. Biosynthesis of choline derivatives. More detail information including all metabolites involved in the shown processes is provided in S5 Fig. Hv acid—homovanillic acid; PhPrP—Phenylpropanoid Pathway; SAR—systemic acquired resistance; PPP—Pentose phosphate pathway.G6P –glucose 6-phosphate; 3PGA– 3-phosphoglyceric acid; PEP—phosphoenolpyruvic acid; G1P –glucose 1-phosphate; galactose1P –galactose 1-phosphate; glycerol-3P- glycerol 3-phosphate; hv acid—homovanilic acid.

Quantified metabolic data can be explored for the determination of major metabolic differences between distinct wheat varieties as well as metabolic changes caused by *F*. *graminearum* infection. Major metabolite changes following infection in the four studied wheat varieties are shown in Fig 5B. These major metabolic changes can be viewed in the context of metabolic pathways involved in the production of resistance related metabolites (Fig 6 and S7 Fig). In FL62R1 major concentration changes include increase in sugars: stachyose and galactose, possibly indicating changes in the cell wall polysaccharides following infection (Fig 5B). Indication of cell wall structure and production changes is corroborated by large increase in concentration in inositols observed at both 48 and 96 hpi (Fig 6). In fact, in all four varieties there is an increase in concentrations of sugars and inositols suggesting an attempt at creation of cell wall barrier for *F*. *graminearum* penetration in all wheat varieties particularly for Sumai3 and FL62R1. This observation is in agreement with previous works [11,12]. According to SAM analysis, agmatine concentration is significantly altered in FL62R1 (particularly at 96 hpi), suggesting significance of Fig 6C spermine producing pathway (agmatine changes shown in Fig 6 are skewed due to near zero concentration in control samples of Stettler). Spermine, possibly synthesized from arginine via agmatine is hypothesized to act as an inducer of pathogenesis-related (PR) proteins and as a trigger for caspase-like activity [32]. Putrescine, also produced from agmatine, has been suggested as possible source of 4-aminobutyrate (GABA) [33], another known factor of resistance to FHB in wheat. GABA is produced in plants in response to stress, however in this work no major increase in GABA concentration was observed. Number of possible roles of GABA and GABA-shunt (production mechanism bypassing two steps in tricarboxylic acid (TCA) cycle and producing GABA from glutamate) have been listed before, however they need further specification in terms of wheat- *F*. *graminearum* interaction [34]. In the studied wheat varieties, there is some increase in GABA concentration in FL62R1 at 48 hpi and in Sumai3 type at both time points post infection but this is not statistically significant in the small sample set studied and its relevance certainly needs to be further explored.

In Sumai3, the most resistant wheat variety included in this work, larger number of metabolites shows statistically significant concentration increase at 48 and 96 hpi compared to control samples. Once again the level of galactose is significantly increased where, in this case, concentrations of myo-inositol (m-inositol) and scyllo-inositol (s-inositol) where also significantly increased at 48 and 96 hpi indicating changes in the cell wall production (Fig 6A). Shikimate concentration is also significantly increasedindicating upregulation of shikimate and PhPrP pathways (Fig 6B). The concentration of agmatine is once again significantly increased and combined with significant increase in citrulline, suggests the importance of the spermine route (Fig 6C) in resistance of Sumai3. Homovanillic acid is a phenolic compound that can be produced from tyrosine and shikimate. As a phenolic compound it has a role in resistance through the production of salicylate, as an antioxidant or as activator of PR resistance factors. Concentration of homovanillic acid is statistically significantly changed in Sumai3 type but its concentration is also increased to a lesser extent in FL62R1 (Fig 6B).

In all wheat varieties studied here it is interesting to observe a large increase in lactic acid production in FL62R1 and Sumai3 varieties (Fig 5B). Lactate could be a by-product of inefficient glycolysis, it can be induced by hypoxia in plant cells or could be caused by changes in NADH/NAD+ ratio and thus observed lactate increase requires further exploration. It is also interesting to point out to a general increase in amino acid concentrations in most cases, particularly in branched chain amino acids. Branched chain amino acids have many possible roles in plants as alternative precursors of TCA cycle during severe plant stress. In case of sugar starvation branched chain amino acids promote their own catabolism [35] but with concentration increases in both sugars and branched chain amino acids in all four wheat varieties this is clearly not the case in *F*. *graminearum* infection. Interestingly, branched chain amino acids are considered cytotoxic, causing apoptosis in mammalian cells [35]. Apoptosis is a known response to stress in plants thus possibly indicating a role for the observed increase in branched chain amino acids [36]. However, as metabolomics cannot discriminate between sources of observed changes of metabolite concentration can be in some cases attributed to *F*. *graminearum*. Increased biosynthesis of branched chain amino acids has been shown in cells of the model organism Aspergillus nidulans particularly at low glucose levels and under hypoxic conditions. In this case branched chain amino acids were part of regulatory mechanisms for NADH/NA+ and NADPH/NADP+ in fungi [37].

**Fig 7:**
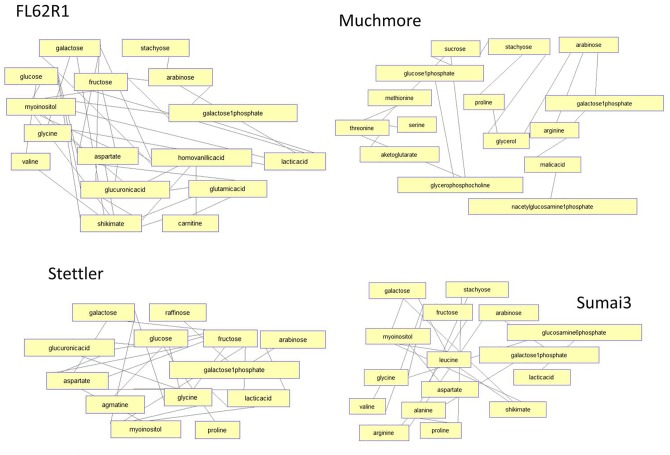
Pearson correlation analysis of metabolic concentration changes over three time points in different wheat varieties. Network shows both positive and negative correlation of over 95%.

Distinct responses in four wheat varieties can be also observed by analyzing correlation networks/clusters of metabolites. Fig 7 shows highly similar correlation (over 95%) of concentration changes across three time points in each wheat variety. For both FL62R1 and Sumai3 there is a related change between carbon sources (sugars), cell wall enhancement metabolites (inositol and galactose) and phenol resistance response factors (shikimate and homovanillic acid). In the most susceptible varieties, Muchmore and Stettler, correlation between concentration changes in sugars and resistance related metabolites is reduced. In resistant varieties, FL62R1 and Sumai3, concentration changes in branched chain amino acid, valine, are highly correlated with changes in sugars and shikimate. This correlation suggests that in the most resistant varieties valine is produced following the Fg inoculation together with shikimate and sugars. In the most susceptible varieties, concentration changes in branched chain amino acids, shikimate and sugars are not correlated suggesting possibly different sources (plant vs. fungus) of observed concentration increase in branched chain amino acids in susceptible varieties. Further analysis needs to explore specific sources of measured metabolites and their relationship with resistance. Analysis of additional time points, plant varieties and environments should substantiate the correlation between the metabolites identified in the present student and type II resistance against FHB. This could lead to the development of robust metabolic markers for FHB resistance.

## Conclusions

Quantitative NMR metabolomics analysis allows biological investigation of metabolic changes in infected plants. SAM analysis of significantly altered metabolites, analysis of relative changes to metabolite concentrations following infection, and correlation analysis of metabolic changes over time indicate activation of resistance pathways in FL62R1 and Sumai3. Activation of cell wall fortification pathways as well changes in amino acid metabolism with particular increase in concentration of branched chain amino acid is observed in all wheat varieties regardless of the level of resistance to FHB. However correlation analysis led to the hypothesis that in resistant varieties branched chain amino acids are produced by wheat and in susceptible varieties they might be result of fungal metabolism. Major changes in the concentration of amino acids, lactic acid as well as galactose and inositols present an interesting route for investigation of function of metabolites in plant—fungus interaction. Sumai3, the most resistant wheat variety studied here, has the fastest and the strongest response primarily through the activation of shikimate and PhPrP pathways with number of metabolites produced in these pathways showing concentration increase. The analysis presented here can pave the way for further application of unbiased, inexpensive, fast and highly reliable NMR methods for the analysis of any plant—pathogen interaction.

## Supporting Information

S1 Fig Data analysis procedure used in the manuscript. (TIF)

S2 Fig Disease severity levels in four studied wheat varieties: Sumai3, FL62R1, Stettler, and Muchmore. These data represent two independent tests that were performed in addition to the one presented in Fig 1. The percentage of infected rachis nodes per head was scored at the time points indicated. Two heads per plant and 20 plants per variety, for a total of 40 heads, were examined for each variety at each time point. Values represent means ± standard error. A one-way ANOVA of data were performed in each time point at α = 0.05 to determine significance among different varieties. Histograms with different letters are statistically different.
(TIF)

S3 Fig A. 1D NMR spectra for 4 wheat varieties at different treatments; B. Basic assignment of major peaks in the 1D spectra.
(TIF)

S4 Fig TOCSY spectra for representative samples for each wheat variety. Spectra show data for control, untreated samples.
(TIF)

S5 Fig Traces of 2D JRES spectra for representative samples for each wheat variety. Spectra show data for control, untreated samples.
(TIF)

S6 Fig Metabolite quantification based on 2D JRES spectra for representative, control samples for each wheat variety. Assignment and quantification of 2D JRES spectra was performed using method provided under Birmingham Metabolite Library (Ludwig et al., 2012).
(TIF)

S7 Fig Wheat primary metabolism and its connections to secondary metabolite production pathways. PhPrP—Phenylpropanoid Pathway; SAR—systemic acquired resistance. SAR—systemic acquired resistance; PPP—Pentose phosphate pathway; gamma-Aminobutyric acid (GABA); G6P –glucose 6-phosphate; 3PGA– 3-phosphoglyceric acid; PEP—phosphoenolpyruvic acid; G1P –glucose 1-phosphate; galactose1P –galactose 1-phosphate; glycerol-3P- glycerol 3-phosphate; hv acid—homovanilic acid.
(TIF)

S1 Table Relative concentrations for metabolites obtained from NMR spectral analysis are presented averaged across biological replicates with standard deviations between replicates. (XLSX)
