# Supplementary figures and images for: Metabolic Biomarker Panels of Response to Fusarium Head Blight Infection in Different Wheat Varieties
Source: PLoS One. 2016 Apr 21;11(4):e0153642. doi: 10.1371/journal.pone.0153642 (PMC4839701; doi:10.1371/journal.pone.0153642)

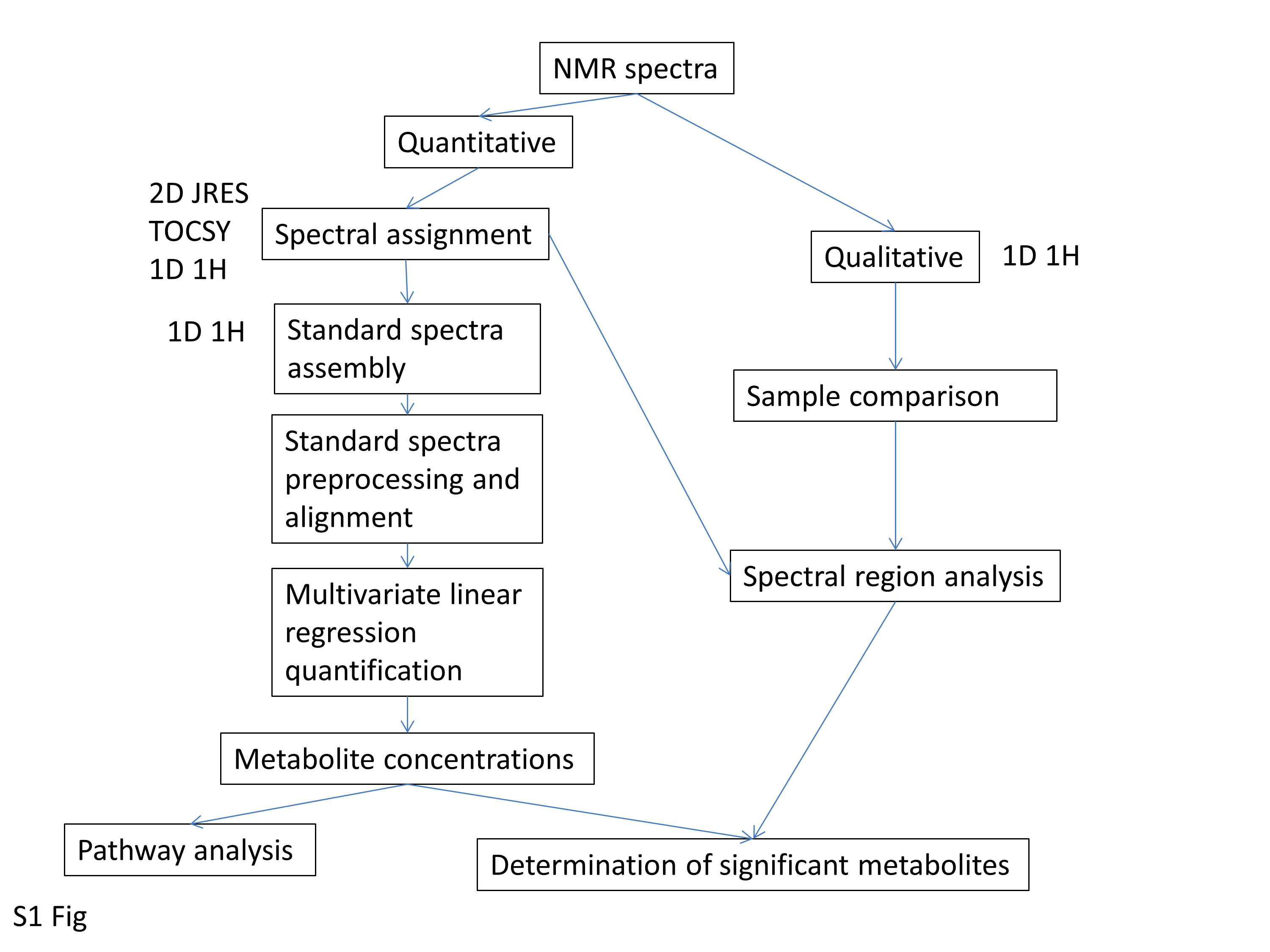

Supplement: S1 Fig — (TIF) [file pone.0153642.s001.TIF]

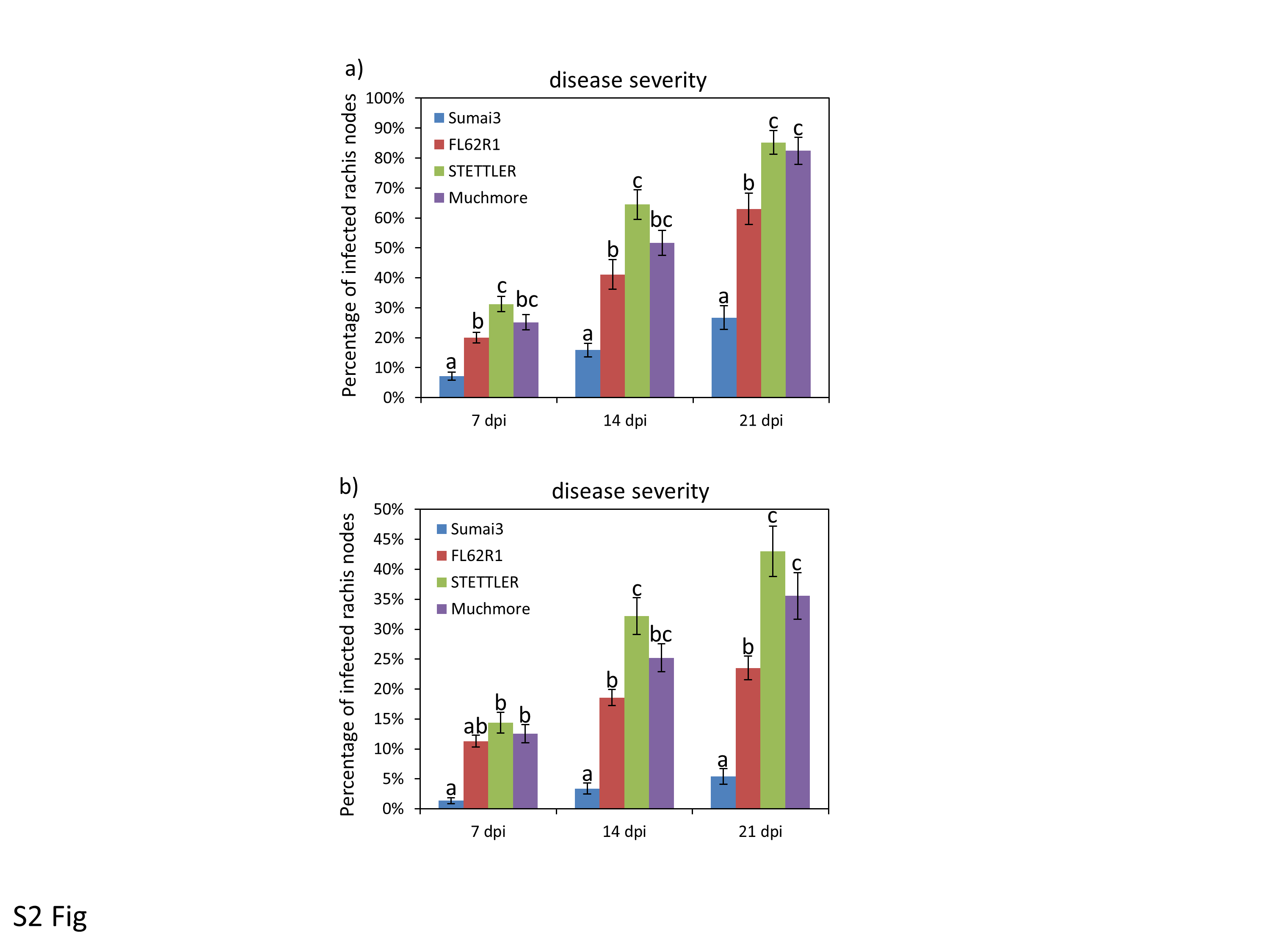

Supplement: S2 Fig — These data represent two independent tests that were performed in addition to the one presented in Fig 1. The percentage of infected rachis nodes per head was scored at the time points indicated. Two heads per plant and 20 plants per variety, for a total of 40 heads, were examined for each variety at each time point. Values represent means ± standard error. A one-way ANOVA of data were performed in each time point at α = 0.05 to determine significance among different varieties. Histograms with different letters are statistically different. (TIF) [file pone.0153642.s002.TIF]

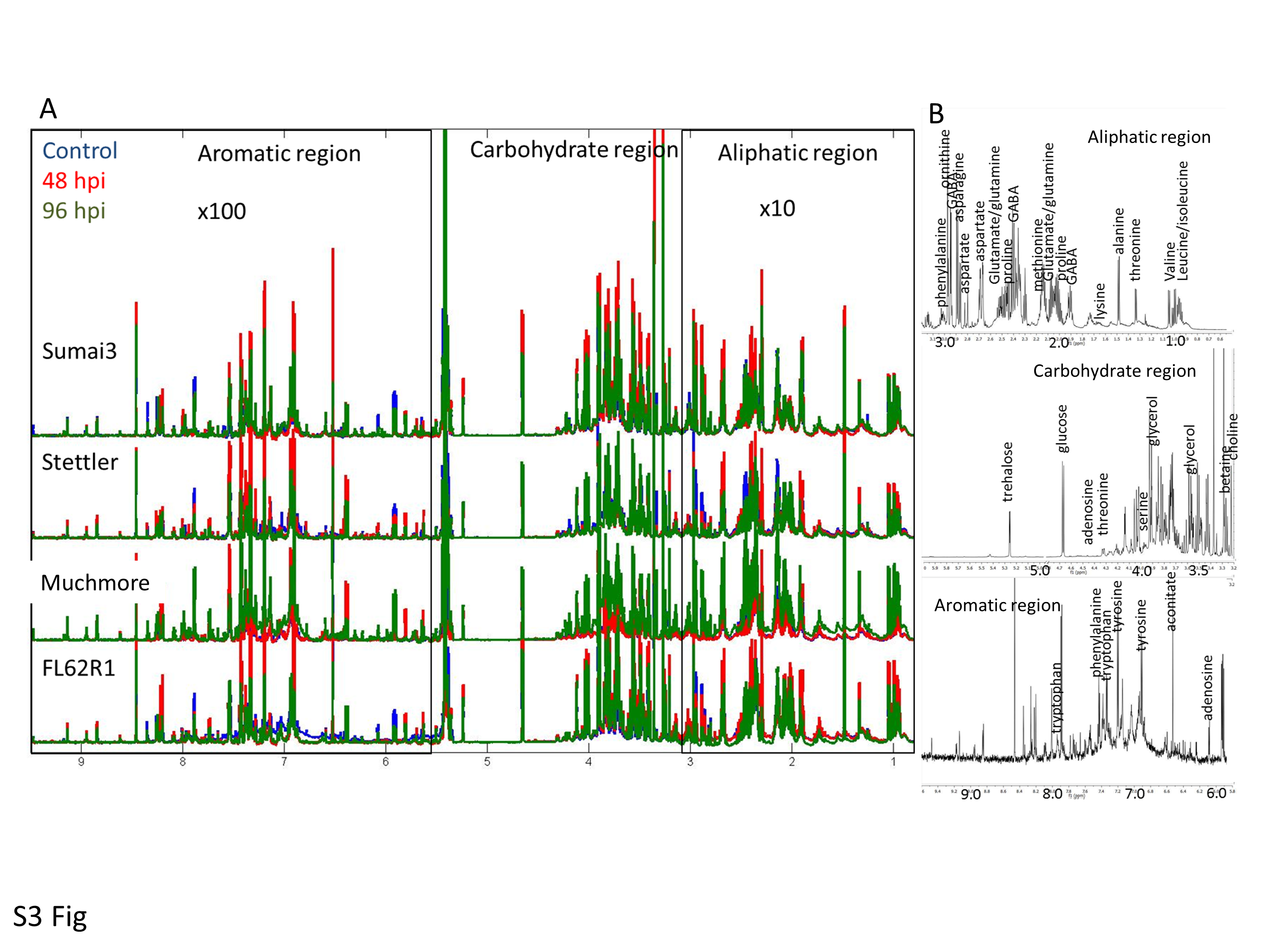

Supplement: S3 Fig — A. 1D NMR spectra for 4 wheat varieties at different treatments; B. Basic assignment of major peaks in the 1D spectra. (TIF) [file pone.0153642.s003.TIF]

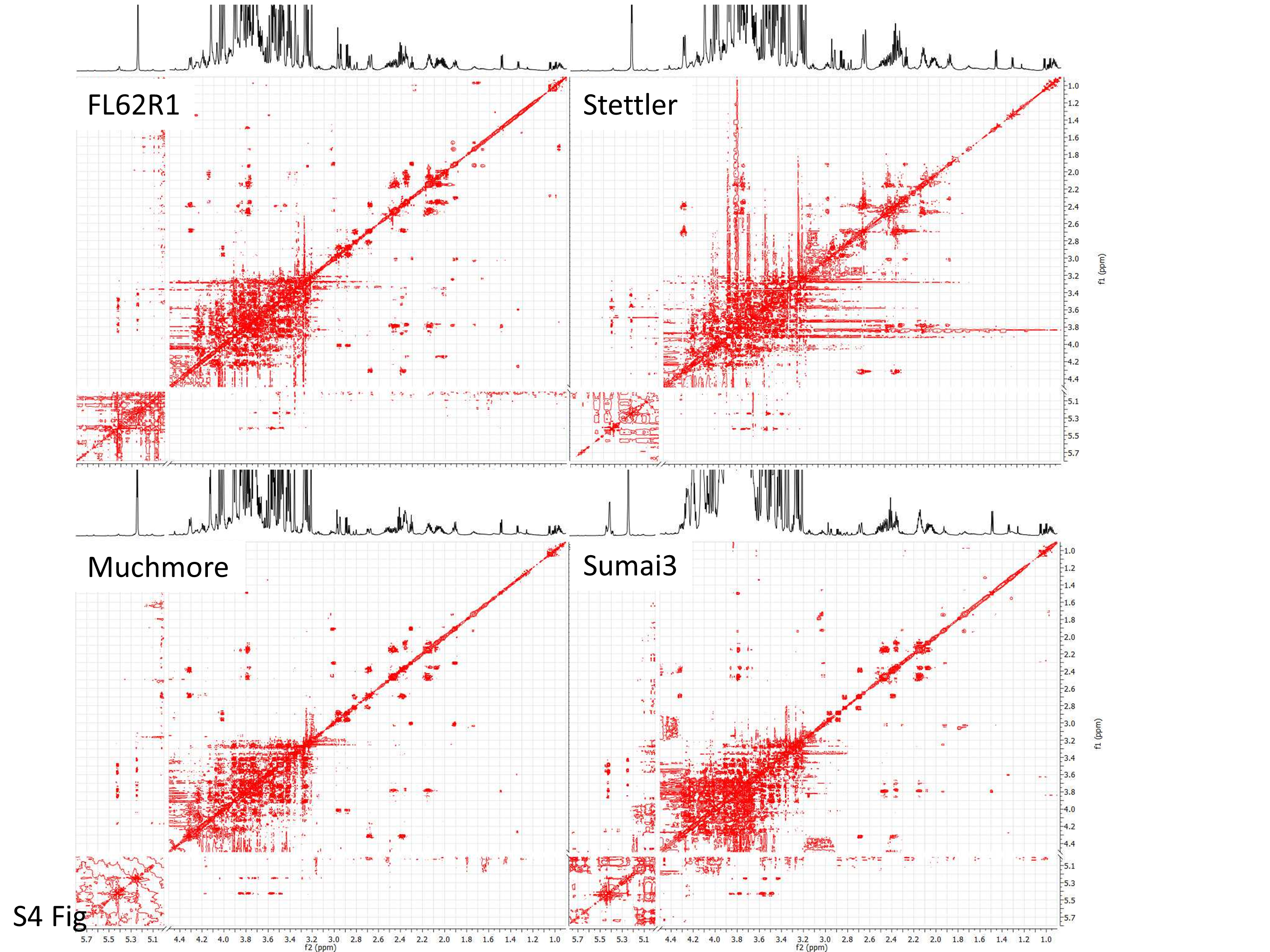

Supplement: S4 Fig — Spectra show data for control, untreated samples. (TIF) [file pone.0153642.s004.TIF]

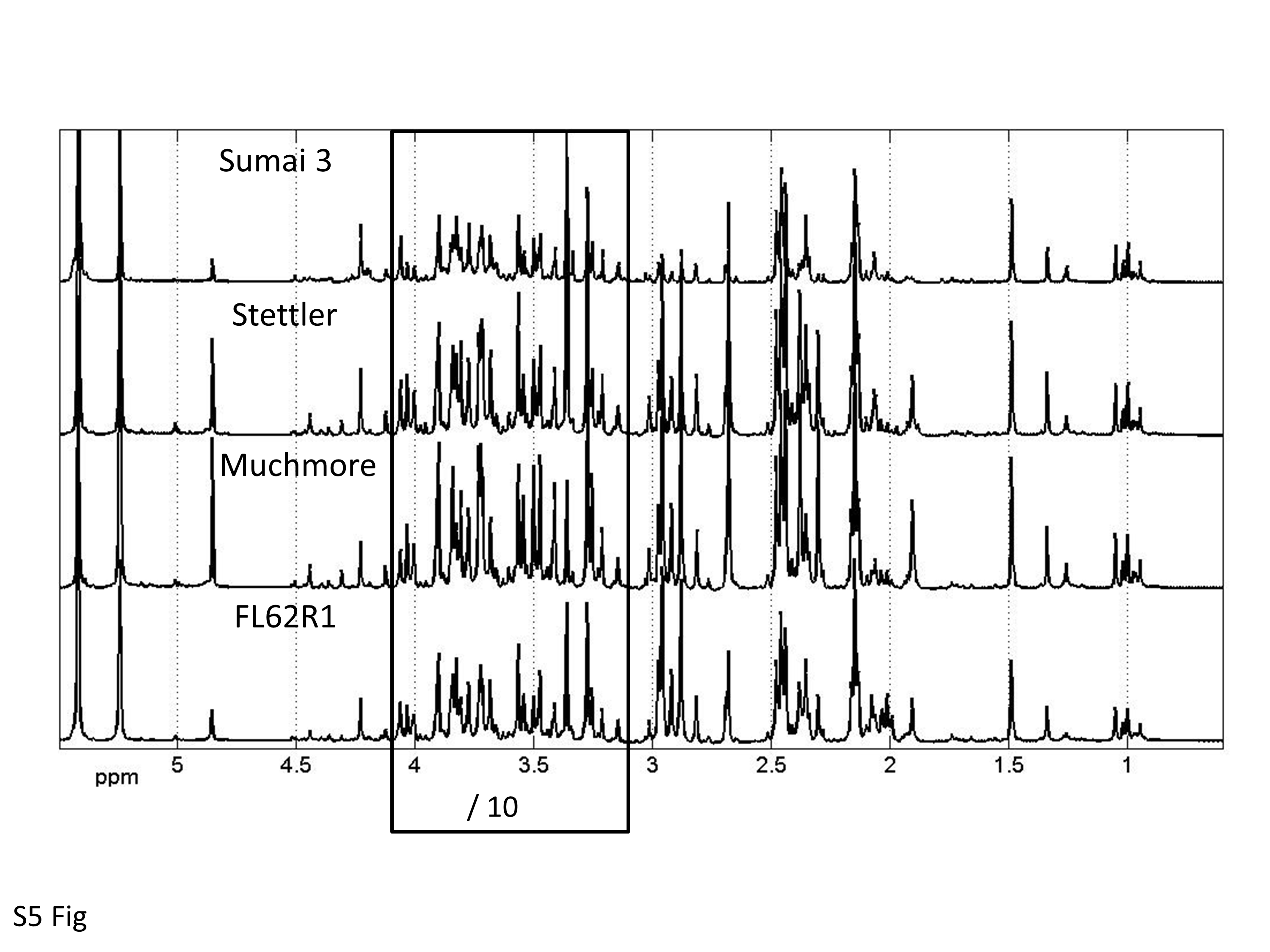

Supplement: S5 Fig — Spectra show data for control, untreated samples. (TIF) [file pone.0153642.s005.TIF]

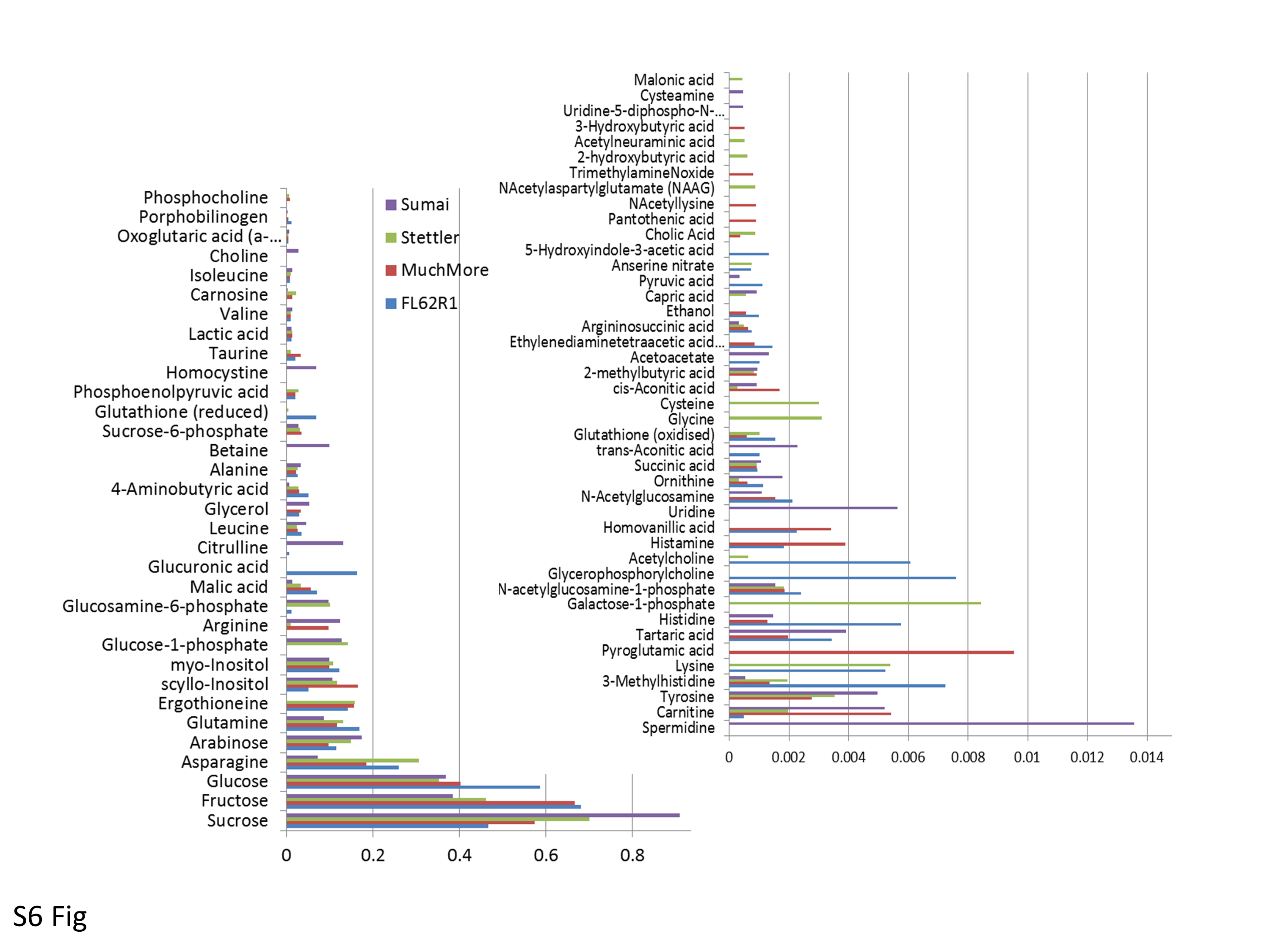

Supplement: S6 Fig — Assignment and quantification of 2D JRES spectra was performed using method provided under Birmingham Metabolite Library (Ludwig et al., 2012). (TIF) [file pone.0153642.s006.TIF]

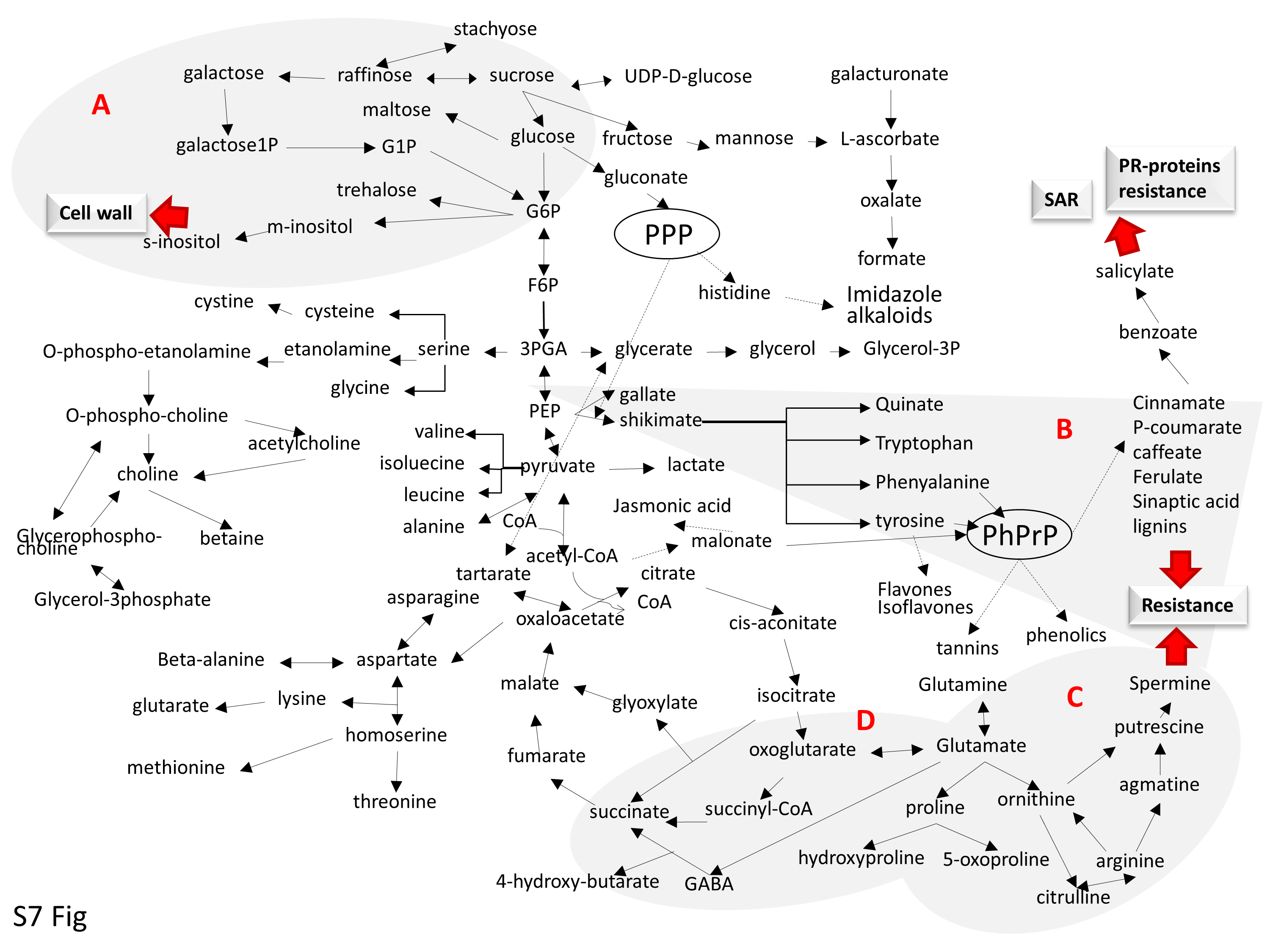

Supplement: S7 Fig — PhPrP—Phenylpropanoid Pathway; SAR—systemic acquired resistance. SAR—systemic acquired resistance; PPP—Pentose phosphate pathway; gamma-Aminobutyric acid (GABA); G6P –glucose 6-phosphate; 3PGA– 3-phosphoglyceric acid; PEP—phosphoenolpyruvic acid; G1P –glucose 1-phosphate; galactose1P –galactose 1-phosphate; glycerol-3P- glycerol 3-phosphate; hv acid—homovanilic acid. (TIF) [file pone.0153642.s007.TIF]
